# Cellular Substrates for Cell-Based Tissue Engineering of Human Corneal Endothelial Cells

**DOI:** 10.7150/ijms.34440

**Published:** 2019-07-21

**Authors:** Qin Zhu, Hong Sun, Dongmei Yang, Sean Tighe, Yongsong Liu, Yingting Zhu, Min Hu

**Affiliations:** 1 Department of Ophthalmology, The Second People's Hospital of Yunnan Province (Fourth Affiliated Hospital of Kunming Medical University); Yunnan Eye Institute; Key Laboratory of Yunnan Province for the Prevention and Treatment of ophthalmology (2017DG008); Provincial Innovation Team for Cataract and Ocular Fundus Disease (2017HC010); Expert Workstation of Yao Ke (2017IC064), Kunming 650021, China; 2 Department of Ophthalmology, the First Affiliated Hospital of Nanjing Medical University, Nanjing, 210029, China; 3 Tissue Tech, Inc., Ocular Surface Center, and Ocular Surface Research & Education Foundation, Miami, FL, 33173 USA; 4 Department of Ophthalmology, Yan' An Hospital of Kunming City, Kunming, 650051, China

**Keywords:** substrate, tissue engineering, endothelium, collagen, integrin, focal adhesion kinase, leukemia inhibitory factor

## Abstract

Corneal endothelial tissue engineering aims to find solutions for blindness due to endothelial dysfunction. A suitable combination of endothelial cells, substrates and environmental cues should be deployed for engineering functional endothelial tissues. This manuscript reviews up-to-date topics of corneal endothelial tissue engineering with special emphasis on biomaterial substrates and their properties, efficacy, and mechanisms of supporting functional endothelial cells *in vitro*.

## Introduction

Corneal endothelial cells are important for visual function by regulating stromal hydration and maintaining corneal transparency. Unfortunately, these endothelial cells are generally not proliferative *in vivo* and cannot replace defective cells. Therefore, any corneal endothelial diseases may cause corneal edema and blindness. At present, the only effective treatment of such blindness requires corneal endothelial transplantation. However, there remains a global shortage of donor corneas with no alternative therapies. Recently with the rise of tissue engineering strategies, new discoveries suggest corneal endothelial progenitors are present in human adult corneal culture. Therefore, it is practical to engineer corneal endothelial grafts *in vitro* in an appropriate environment with appropriate isolation methods, culture substrates, media, and other environmental conditions. In this article, we focus on culture substrates and their ability to support functional endothelial cells *in vitro*.

### Collagen IV

Collagen IV is the primary collagen in extracellular basement membranes separating epithelial and endothelial cells. Since the discovery of collagen IV by Kefalides in 1966, collagen IV has been investigated extensively by numerous research laboratories around the world. So far, six mammalian genes encoding six polypeptide chains of collagen IV α-chain polypeptides (α1-α6) have been discovered and subsequently characterized (reviewed in 1). The NC1 domain is critical for the trimeric structure of the type IV collagen.

### Known Functions of Collagen IV

Type IV collagen filaments are linked to interstitial collagen fibers and endothelial basement membranes 2. Collagen IV is a critical mediator of cell behavior 3, tissue compartmentalization, the external microenvironment 3, blood vessel maintenance, and responses to extracellular microenvironment sensors in endothelial cells and pericytes 1.

Collagen IV has been idenfitied to be a key basement membrane collagen in endothelial and epithelial layers 4, suggesting collagen IV is critical for endothelial structure and functions. It is likely collagen IV maintains the normal phenotype of human corneal endothelial cells (HCECs) and prevents endothelial mesenchymal transition (EMT). For example, bovine corneal endothelial cells lose their phenotype with increased α-smooth muscle actin expression and formation of fibronectin fibril assembly when seeded on glass or tissue culture polystyrene. Bovine corneal endothelial cells also lose expression of ZO-1 when seeded on fibronectin and collagen I. However, when seeded on collagen IV, the endothelial cells are morphologically and phenotypically similar to in vivo state with polygonal shape and ZO-1 expression located borderly and F-actin located cortically 5, indicating that collagen IV plays a critical role in maintaining endothelial phenotype. On collagen IV coated dishes, HCECs also maintain higher cell densities with polygonal shape 6 (also reviewed in 7) with greater attachment 7, 8. Consistent with the notion that Collagen IV is an important substrate, it had been shown normal endothelial cells secrete collagen IV while fibroblastic corneal endothelial cells mainly secrete collagen I 9.

We have screened different substrates such as collagen IV, matrigel, laminin and fibronectin that can be coated on an atelocollagen carrier for engineering HCEC grafts and noted that collagen IV is the most ideal substrate to be used to coat the atelocollagen carrier for expansion of HCECs 10. Because collagen IV is the best substrate for culturing HCECs, all our experiments have been performed with collagen IV-coated dishes or atelocollagen sheets. Despite the known importance of Collagen IV, it remains unclear of the mechanism of action in how it promotes cell attachment and growth on atelocollagen sheets. It also remains unclear how collagen IV may affect the behavior of HCEC aggregates (not single cells) such as phenotype on plastics 10-18 on atelocollagen sheets.

### Atelocollagen

Atelocollagen is a derivative of collagen I obtained by removal of N- and C-terminal telopeptide components. Because atelocollagen is solubilized by protease, its physical properties are virtually identical to those of natural, unsolubilized collagen. In addition, atelocollagen has little immune antigenicity as it is composed of a G-X-Y amino acid sequence that differs little even among different animal species. The slight amount of antigenicity in collagen is due to the telopeptides attached tocollagen molecules without G-X-Y sequence.Although such collagen may resist immune-rejection, it may also not support cell attachment and expansion.

### Integrins

Integrins are composed with two subunits, that is, α and β subunits. Integrins form complexes with matrix proteins including collagens, fibronectin and laminins 19. Integrins signal through their receptors, which are important for endothelial cells to attach to the extracellular matrix, and are mediated by various α and β integrin subunits. For example, the attachment of endothelial cells to fibronectin is mainly through α4β1 and α5β1 integrins, while their attachment to laminin is mainly through α3β1, α6β1 and α6β4 integrins 20. In angiogenesis, incorporation of integrin αvβ3 with collagen IV mediates endothelial cell adhesion, migration and proliferation 21-23. Inhibition of collagen IV production by cis-hydroxyproline reduces tube formation, while augmentation of exogenous collagen IV promotes tube formation 24. Integration of collagen IV with integrin αvβ3 from endothelial cells may result in activation of integrin-mediated signaling in endothelial cells 21, 22. Such integrin activation may inhibit apoptosis in pulmonary vascular endothelial cells induced by LPS 25, 26.

However, it remains unclear whether collagen IV binds to integrin in our endothelial models and activates integrin-mediated signaling?

### Interaction of Integrins and Collagen IV

Collagen IV is crucial for the appropriate interaction of cells with the basement membrane including cell adhesion, proliferation, differentiation and migration 27, 28. In fact, collagen IV is an important binding substrate for numerous cell types, for example, endothelium 29, hepatocytes 30, keratinocytes 31, mesangial cells 32, pancreatic cells 33, platelets 34, 35, and tumor cells such as breast and prostate carcinoma 36, 37, melanoma 27 and sarcoma 38.

The major integrins includes β_1_ integrins, for example, α_1_β_1_ and α_2_β_1_ 39-41. Integrin α_1_β_1_ has a high affinity for collagen IV, while α_2_β_1_ perfers collagen I 42, 43. Deletion of α_1_β_1_ integrin may cause significant reduction in adhesion and migration of fibroblasts and adhesion of smooth muscle cells to collagen IV 44. Functional activity of α_1_β_1_ integrin has been demonstrated by synthetic peptide with 12 amino acid residues (457-468) from collagen IV 45. Nontheless, collagen IV has been shown to bind with α_2_β_1_ integrin 46 and α_3_β_1_ integrin 47-50.

Specific binding sites of integrins have been identified for α3 NC1 domain 51, 52. For example, residues 54-132 of α3 NC1 domain is associated with apoptosis and reduced tumor growth in mice 53. Another binding site was at position 185-203 of α3 NC1 domain which resulted in inhibition of melanoma cell proliferation 51, 54, 55. However, it remains unclear what the predominant downsteam signaling mechanisms of integrin are and, how activation of integrin can affect cell proliferation and phenotype in an endothelial system.

### Focal Adhesion Kinase

Focal adhesion kinase (FAK) is a cytoplasmic tyrosine kinase that is critical for embryonic development and the etiology of human diseases 56, 57. FAK is also widely expressed in many tissues and has three major functions:motility, survival and proliferation. Integrin-dependent FAK signaling is critical for survival 58, 59. FAK also plays an important role in mediation of adhesion responsive elements to promote proliferation and activate transcription factors 60, 61. FAK also regulates actin cytoskeleton, thus, mediating cell motility 62.

FAK has 4 domains, N-terminal FERM domain, catalytic tyrosine kinase domain, C-terminal focal-adhesion targeting (FAT) domain and proline-rich region not specified. Integrin-mediated adhesion activates FAK by phosphorylating tyrosine 397, resulting in formation of a binding site for Src-homology 2 (SH2) of Src, which then phosphorylates other tyrosine sites in FAK and thus amplifies its kinase activity dramatically 63. Activation of FAK-Src complex promotes Rac1 activity via phosphorylation of the scaffolding p130Cas protein ( Bcar1) 64. Such phosphorylation enhances recruitment of Dock180 and motility 1 (ELMO1), which functions as a GEF for Rac1 to promote membrane protrusions 65, 66. FAK-Src complex can also phosphorylate paxillin, recruiting the ArfGAP paxillin-kinase linker (PKL) and Pak-interacting exchange factor-beta (β-PIX), activating Rac1 via a direct interaction 67. Interestingly, PKL and β-PIX may be phosphorylated through Src, regulating their activities in integrin-mediated adhesion 68, 69.

### FAK Signaling Interacts with STAT3 Signaling to Promote Cell Growth

Previous publications have suggested that v-Src activation inhibits apoptosis and promotes anchorage-independent growth through activation of PI 3-kinase and STAT3 (pY705) signalings 70-74. Activated FAK signaling can also activate STAT3 (pY705) to facilitate anchorage-independent growth 75. Conversely, we have also reported that LIF-JAK-STAT3 (pY705, LIF, leukemia inhibitory factor) signaling promotes HCEC growth by delaying contact-inhibition 17. Activated LIF may promote JAK-STAT3 (pY705) signaling 76. It is unclear whether activation of FAK signaling requires potentiation of LIF-JAK-STAT3 (pY705) signaling for promoting HCEC attachment and growth on collagen IV coated dishes/atelocollagen sheets, and if so how the two signalings interact. STAT phosphorylation at Y705 position may be the key for survival of HCECs on atelocollagen sheets coated with collagen IV.

LIF may induce various cellular responses, for example, differentiation, proliferation 77, and embryogenesis 78, 79. LIF is also a key cytokine for sustaining self-renewal and pluripotency of mouse ESCs and iPSCs. Upon binding to its receptor (R), LIF-R stimulates activation of signal transducer glycoprotein 130 (gp130), which then activates gp130-associated JAK kinases 80, 81. Activated JAK kinases phosphorylates STAT3 proteins (pY705-STAT3), promoting JAK/STAT (pY705) signaling. When phosphorylated, the STAT3 proteins are dimerized, going into the cell nucleus to mediate expression of targeted genes 82. Thus, STAT3 is a key mediator downstream of LIF. In the JAK family, JAK1 and JAK2 are closely linked to LIF signaling 83. JAK1 is also critical for self-renewal of murine ESCs 84. These suggest activation of LIF-JAK1-STAT3 (pY705) signaling may be involved in delaying contact inhibition and over-expression of ESC and NC markers of HCEC monolayers in modified embroyonic stem cell media (MESCM). In fact, we have discovered that LIF, but not bFGF, in MESCM plays a pivotal role in delaying contact inhibition of HCEC monolayers in the late phase (D35) of culture 17. Further analysis indicates that such delaying contact inhibition is associated with upregulated expression of positive G1/S phase transition genes by activating LIF-JAK1-STAT3 signaling pathway 17. In such an event, the signaling is via phosphorylation of tyrosine 705*.* If *Stat3 (pY705)* is lost, embryonic mice may not survive 85. *Stat3 (pY705) also mediates* cell proliferation, apoptosis in numerous tissues 86, and self-renewal of embryonic stem cells 76, 87. However, its role and mode of action during neural crest formation remains largely unknown.

In contrast, STAT3 (pS727) may just play a minor role in cellular biological process. In this process, STAT3 proteins may be phosphorylated at serine 727 (S727) through mitogen-activated protein kinases (MAPK) and c-Jun kinases 88-90. However, such interactions between MAPK and STAT3 (pS727) are not well understood. STAT3 (pS727) plays an important role for maximized function of the gene transcription and for promotion of the cell growth in vitro 91, probably synergestically with STAT3 (pY705). Interestingly, integrin-mediated FAK signaling mediates mitochondrial bioenergetics, probably through nuclear translocation of pS727-STAT3 92. Such signal is important for actin reorganization, cell mobility, cell adhesion, and cell cycle mediation 93. When activated, STAT3 may translocate due to S727 cytoplasmic phosphorylation 94. Integrin-activated FAK signaling via STAT3 (S727) can decrease ATP synthesis, which is key to prevent mitochondrial dysfunction, apoptosis, and subsequent cell death 95. It remains unclear whether the integrin-FAK-STAT3 pathway activated by collagen IV plays the same or different roles in HCECs. It is also unclear how FAK activates STAT3 (pS727). And if so, how such activation of STAT3 (S727) affects the attachment and proliferation of HCECs on atelocollagen sheets coated with collagen IV. And if so, whether such activation of STAT3 (S727) inhibits apoptosis of HCECs on atelocollagen sheets coated with collagen IV, and if so, via which integrin?

## Conclusion

In the past few decades, major efforts has been invested in developing tissue engineering techniques. One of the main strategies for effective tissue engineering is the proper selection of the cell substrates. For human corneal endothelial engineering, the methods are conditioned to the need of human corneal endothelial growth and with an environment which resembles the cellular and environmental conditions in vivo. Overall these elements are critical for successful engineering of functional tissue with normal phenotype and genotype.
